# Reliability and Validity of Wisconsin Upper Respiratory Symptom Survey, Korean Version

**DOI:** 10.2188/jea.JE20100160

**Published:** 2011-09-05

**Authors:** Su-Young Yang, Weechang Kang, Yoon Yeo, Yang-Chun Park

**Affiliations:** 1 Department of Internal Medicine, Oriental Medical College of Daejeon University, Daejeon, South Korea; 2 Department of Business and Information Statistics, Economics College of Daejeon University, Daejeon, South Korea; 3 College of Pharmacy, Purdue University, West Lafayette, IN, USA; 4 Weldon School of Biomedical Engineering, Purdue University, West Lafayette, IN, USA

**Keywords:** quality of life, Wisconsin Upper Respiratory Symptom Survey, reliability, validity

## Abstract

**Background:**

The Wisconsin Upper Respiratory Symptom Survey (WURSS) is a self-administered questionnaire developed in the United States to evaluate the severity of the common cold and its reliability has been validated. We developed a Korean language version of this questionnaire by using a sequential forward and backward translation approach. The purpose of this study was to validate the Korean version of the Wisconsin Upper Respiratory Symptom Survey (WURSS-K) in Korean patients with common cold.

**Methods:**

This multicenter prospective study enrolled 107 participants who were diagnosed with common cold and consented to participate in the study. The WURSS-K includes 1 global illness severity item, 32 symptom-based items, 10 functional quality-of-life (QOL) items, and 1 item assessing global change. The SF-8 was used as an external comparator.

**Results:**

The participants were 54 women and 53 men aged 18 to 42 years. The WURSS-K showed good reliability in 10 domains, with Cronbach’s alphas ranging from 0.67 to 0.96 (mean: 0.84). Comparison of the reliability coefficients of the WURSS-K and WURSS yielded a Pearson correlation coefficient of 0.71 (*P* = 0.02). Validity of the WURSS-K was evaluated by comparing it with the SF-8, which yielded a Pearson correlation coefficient of −0.267 (*P* < 0.001). The Guyatt’s responsiveness index of the WURSS-K ranged from 0.13 to 0.46, and the correlation coefficient with the WURSS was 0.534 (*P* < 0.001), indicating that there was close correlation between the WURSS-K and WURSS.

**Conclusions:**

The WURSS-K is a reliable, valid, and responsive disease-specific questionnaire for assessing symptoms and QOL in Korean patients with common cold.

## INTRODUCTION

The “common cold” is caused by a viral infection of the upper respiratory tract.^1^ This syndrome is generally self-limiting with time, but its symptoms negatively influence quality of life (QOL), and its prevalence imposes huge social and economic burdens.^2^ In Korea, upper respiratory tract infection is the most common reason for outpatient visits.^3^ In addition, such infections can result in complications, such as secondary bacterial infections, exacerbation of asthma, and chronic obstructive airway diseases.^4^^,^^5^

A number of clinical trials have investigated the common cold.^6^^–^^10^ Cellular immune responses, antibody titers, and lymphocyte proliferation have been used for quantitative assessment of the common cold in these studies.^9^^–^^12^ However, these are not suitable for this purpose because the common cold is caused by various viruses, including rhinovirus, which have more than 100 different serotypes.^13^ Thus, it is difficult to find specific biomarkers of progression of common cold. For this reason, symptom change is considered a standard indicator of the effect of cold remedies in many clinical studies.^14^ However, if symptom change is to serve as a reliable indicator of common cold in clinical studies, it is critical that quantitative scales of such change are established and validated.

The Wisconsin Upper Respiratory Symptom Survey (WURSS), developed by Barrett et al,^15^ is a patient-oriented instrument that evaluates patient QOL in an illness-specific manner. Other scale systems, such as the Jackson Scale^16^ for the common cold, include only a limited number items regarding symptoms^17^^–^^19^ and have rarely been evaluated with respect to their validity.^16^^,^^20^ In contrast, the WURSS includes questionnaires regarding global severity, symptoms, functional QOL, and global change^15^ and has been verified for responsiveness, reliability, and validity through monitoring of participants with common cold.^21^ The WURSS has been widely used in respiratory disease research^11^^,^^22^^–^^24^ and is now accepted as a useful tool in clinical trials of common cold. Because there is no Korean equivalent of the WURSS, we translated the WURSS into the Korean (WURSS-K) and evaluated the validity and reliability of this instrument in patients with upper respiratory tract infection.

## METHODS

### The WURSS and development of the Korean version

The WURSS includes 44 items: 1 global severity item, 32 symptom-based items, 10 functional QOL items, and 1 global change item. All items are based on 7-point Likert-type severity scales.^21^ The content of the WURSS is summarized in Table 1.

**Table 1.** Content of the Wisconsin Upper Respiratory Symptom Survey (WURSS)

Symptoms^a^	Symptoms	Symptoms	Functional impairments^b^
1. How sick do you feel today?	12. Body aches	23. Swollen glands	34. Think clearly
2. Cough	13. Feeling “run down”	24. Plugged ears	35. Speak clearly
3. Coughing stuff up	14. Sweats	25. Ear discomfort	36. Sleep well
4. Cough interfering with sleep	15. Chills	26. Watery eyes	37. Breathe easily
5. Sore throat	16. Feeling feverish	27. Eye discomfort	38. Walk, climb stairs, exercise
6. Scratchy throat	17. Feeling dizzy	28. Head congestion	39. Accomplish daily activities
7. Hoarseness	18. Feeling tired	29. Chest congestion	40. Work outside the home
8. Runny nose	19. Irritability	30. Chest tightness	41. Work inside the home
9. Plugged nose	20. Sinus pain	31. Heaviness in chest	42. Interact with others
10. Sneezing	21. Sinus pressure	32. Lack of energy	43. Live your personal life
11. Headache	22. Sinus drainage	33. Loss of appetite	44. Compared to yesterday, I feel…

^a^Directions for symptom-based items (2–33) ask respondents to “Please rate the average severity of your cold symptoms over the last 24 hours.”

^b^Directions for items on functional impairment (34–43) ask: “Over the last 24 hours, how much has your cold interfered with your ability to…?”

Reprinted from Barrett et al. “The Wisconsin Upper Respiratory Symptom Survey is responsive, reliable, and valid.” *J Clin Epidemiol* 2005;58(6):609–17 with permission from Elsevier and the authors.

The first step in developing the WURSS-K was forward translation: a physician bilingual in English and Korean translated the WURSS into Korean. Then, a translation panel composed of internists, an English linguist, a Korean linguist, and a statistician reviewed the translation and prepared the first Korean draft version (WURSS-K version 1.0). The second step was backward translation: another bilingual physician, blinded to the WURSS, back-translated the WURSS-K version 1.0 into English, and then the panel compared the back-translated English version with the WURSS to identify any ambiguities or inaccuracies in the choice of vocabulary. During this step, we were particularly careful to retain the meaning of the original items, maintaining the subtle differences between descriptions such as “feeling run down”/“feeling tired” and “chest congestion”/“chest tightness”/“chest heaviness.” The second draft version (WURSS-K version 1.1) was prepared after this step. Finally, a cognitive briefing was performed to test the comprehensibility of the WURSS-K version 1.1. Here, the translation panel interviewed 10 patients (age 21–58 years; 50% male) and determined if Korean words for “sinus” and “swollen glands” could be described by patients in layman terms. After completing the above 3 steps, the WURSS-K was submitted for statistical validation (described below) to evaluate its reliability, validity, and responsiveness. The WURSS-K results were compared with those of the WURSS.^22^

### Study design

The Daejeon Oriental Hospital Institutional Review Board approved the protocol (authorization number: DJOMC-19), and all patients understood the purpose and method of this study and provided written informed consent to participate in this research. Participants were recruited from the 3rd to 21st of March 2008 (19 days) in the Daejeon Oriental Hospital of Daejeon University and the Health Center of Daejeon University.

Participants satisfying the following criteria were included in the study: a diagnosis of “common cold” by research physicians, with onset of symptoms within 48 hours. Exclusion criteria included age younger than 18 years or older than 60 years, allergic rhinitis, asthma, chronic obstructive pulmonary disease, sinusitis recurring more than twice per year, anatomical nasal obstruction or deformity, otitis, and exudative pharyngitis.

**Figure 1.**
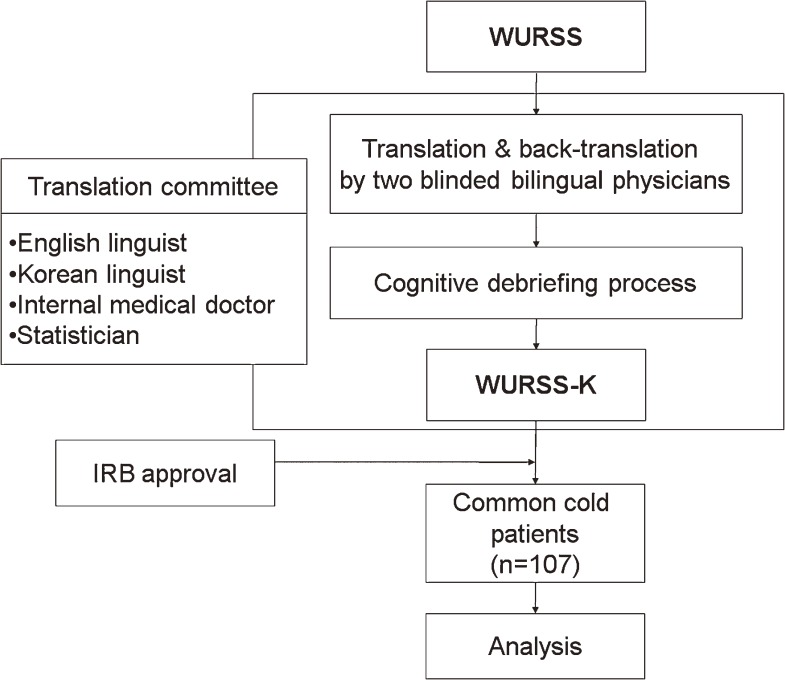
Study design. WURSS-K, Wisconsin Upper Respiratory Symptom Survey, Korean version; IRB, institutional review board.

Participants were asked to complete the WURSS-K daily from the first day for 6 days and were examined with the SF-8 on the third day. The participants were allowed to take cold medicines during this study (Figure 1).

At least 30 cases were required to ensure normality of the estimators in each item of the WURSS-K. The number of participants was calculated based on this requirement. The occurrence rate of each item was estimated from the results of the WURSS, where the lowest occurrence rate was reported to be 36.9% for the item “sweats.”^21^ Therefore, at least 82 (= 30/0.369) participants were required. A total of 107 patients were enrolled to account for potential dropouts during the study period.

### Statistical analysis

Data are reported as mean ± standard deviation (SD) unless otherwise specified. The level of significance for all tests was set at *P* < 0.05. According to the study of the WURSS,^21^ 42 items were classified into 10 domains, except for 1 global severity item and 1 global change item. The internal reliability was evaluated by calculating the Cronbach’s alpha coefficient in each domain based on the average of the scores for the first three days. The Pearson correlation between coefficient alphas of the WURSS-K and reliabilities of the WURSS^21^ was calculated to evaluate coherence in the variety of the internal reliabilities of these 2 questionnaires. Convergent validity was evaluated by Pearson correlation coefficients between total WURSS-K scores observed on the first day and the SF-8 observed on the third day. According to the principle of convergent validity,^25^ a high correlation with other instruments measuring the same concept is required to prove the validity of different instruments measuring the same concept. We used the last 4-week recall SF-8 Korean version on the third day because other Korean versions of the SF-8, ie, 1-week recall and 24-hour recall, were not available. Here, the subjects were required to perform 4-week recall regarding health-related QOL on the third day, ie, around the middle of this study, which was a reasonable time point for assessing the burden of cold symptoms in patients. For the correlation between the WURSS-K and SF-8, we used the WURSS-K score form the first day because it was more likely to influence the last 4-week recall of the SF-8 than scores from other days.

Guyatt et al^26^ suggested that the most appropriate indicator of responsiveness should be able to relate variability in test scores of stable subjects to clinically important changes. Responsiveness of each item on the WURSS-K (except for the 44th item) was evaluated by Guyatt’s responsiveness index ($\mathrm{Responsiveness Index}=\frac{\text{MID}}{\sqrt{2\text{MSE}}}$).^27^ Here, MID indicates minimal important difference, namely, the mean value of day-to-day changes in each item corresponding to assessments of global change of “a little better” or “somewhat better.” MSE is the mean squared error of the scores of stable subjects reporting “the same” on item 44.

The Pearson correlation coefficient was also calculated between responsiveness indices of the WURSS-K and WURSS.

## RESULTS

### Demographics of participants

A total of 107 participants were initially enrolled in the study. One participant changed his mind, and 7 participants did not visit the center. All participants who replied once or more were included in the analysis. Most participants were university students, and age ranged from 18 to 42 years (mean 21.38). There were 54 female and 53 male participants (Table 2).

**Table 2.** Participant characteristics

Variable	Value
Age, years	
Mean	21.38 ± 2.7
Range	18–42
Sex, no./total (%)	
Female	54/107 (50.5)
Male	53/107 (49.5)
Height (mean ± SD), cm	168.9 ± 7.9
Weight (mean ± SD), kg	62.7 ± 10.7
Blood pressure (mean ± SD), mm Hg	
Systolic blood pressure	125.6 ± 15.0
Diastolic blood pressure	73.9 ± 9.5
Pulse (mean ± SD), frequency/min	81.5 ± 13.1
Temperature (mean ± SD), °C	36.3 ± 0.5

### Reliability test

As for internal reliability, the Cronbach’s alpha of the 10 domains (based on the average of scores for the first 3 days) ranged from 0.666 to 0.962. According to guidelines for interpreting coefficient alpha, 0.65 to 0.70 is minimally acceptable, 0.70 to 0.80 is respectable, and 0.80 to 0.90 is very good.^28^^,^^29^ For a coefficient alpha greater than 0.90, it is recommended to consider shortening the scale by reducing the number of items.^28^^,^^29^ The domains with a coefficient alpha of >0.90 were “activity and function,” which was composed of 10 items (0.962); “chest,” with 3 items (0.950); and “ears,” with 2 items (0.935). These domains also had reliabilities greater than 0.90 in the WURSS study.^21^ The reliability coefficients of the WURSS ranged from 0.624 to 0.934. The Pearson correlation between coefficient alphas of the WURSS-K and reliabilities of the WURSS^21^ was 0.710 (*P* = 0.02; Table 3). These results indicate that the internal reliability of the WURSS-K was very similar to that of the WURSS.^21^

**Table 3.** Domains and Reliability of the WURSS-K and WURSS

Domain	Reliability^a^	

	WURSS-K	WURSS
Cough	0.828	0.828
Sore throat	0.865	0.748
Nasal	0.754	0.717
Sinus	0.803	0.872
Aches	0.666	0.624
Tiredness	0.845	0.937
Sweats	0.838	0.799
Ears	0.935	0.916
Chest	0.950	0.912
Activity and function	0.962	0.934

Correlation coefficient^b^	0.710*	

Items measuring global severity and global change on the WURSS-K were not included in the assessment.

^a^Cronbach’s alpha.

^b^Correlation coefficient between WURSS-K and WURSS.

*P = 0.02.

### Validity test

**Figure 2.**
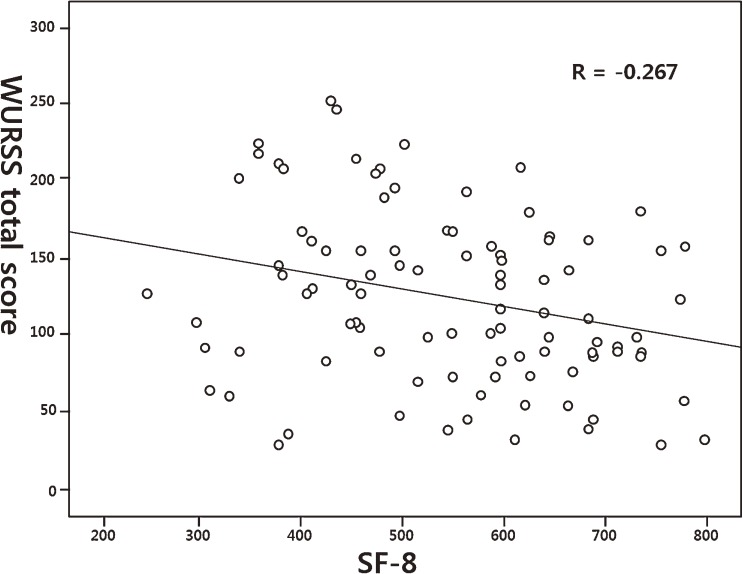
Correlation between the WURSS-K (Wisconsin Upper Respiratory Symptom Survey, Korean version) and SF-8. The WURSS-K included data from day 1.

Convergent validity was evaluated by correlating the total scores of the SF-8 and the total scores on the first day of the WURSS-K. The Pearson correlation coefficient was −0.267 (*P* = 0.007). The WURSS study^21^ used a 24-hour recall version of the SF-8 and observed it from, at a minimum, day 2 through day 5. The correlation coefficients for the WURSS data from day 2 through day 5 were −0.60 to −0.84 (*P* < 0.001), much higher than that of this research (Table 4, Figure 2).

**Table 4.** Coefficient of correlation with SF-8

Survey	Correlation coefficient	*P* value
WURSS-K^a^	−0.267	*P* = 0.007
WURSS^b^	−0.60 to −0.84	*P* < 0.001

^a^SF-8 (4-week recall) was measured on the third day.

^b^SF-8 (24-hr recall) was measured every day.

### Responsiveness test

The responsiveness of each item of the WURSS-K was checked by calculating the Guyatt’s responsiveness index.^26^^,^^27^ The responsiveness indices ranged from 0.13 to 0.46: the maximum index of 0.46 was obtained for the first question, “How sick do you feel today?”, and the minimum index of 0.13 was obtained for the question on “sinus pressure near the nose.” In the WURSS study,^21^ the indices ranged from 0.139 for the question on “plugged ears” to 0.709 for the first question. The correlation coefficient between the responsive indices of the WURSS-K and those of the WURSS^21^ was 0.534 (*P* < 0.001; Table 5), which showed that the responsiveness of these 2 instruments was definitely correlated.

**Table 5.** Responsiveness of WURSS-K and WURSS

Item	Responsiveness^a^	

	WURSS-K	WURSS
1	0.460	0.709
2	0.294	0.300
3	0.224	0.193
4	0.234	0.247
5	0.367	0.278
6	0.324	0.307
7	0.254	0.276
8	0.363	0.370
9	0.318	0.337
10	0.334	0.285
11	0.345	0.258
12	0.267	0.216
13	0.308	0.376
14	0.258	0.152
15	0.287	0.188
16	0.343	0.202
17	0.322	0.120
18	0.332	0.392
19	0.293	0.240
20	0.169	0.183
21	0.128	0.200
22	0.234	0.207
23	0.149	0.203
24	0.165	0.139
25	0.169	0.174
26	0.255	0.191
27	0.253	0.148
28	0.366	0.290
29	0.277	0.226
30	0.184	0.183
31	0.206	0.179
32	0.380	0.323
33	0.217	0.241
34	0.233	0.265
35	0.188	0.204
36	0.347	0.300
37	0.222	0.276
38	0.196	0.416
39	0.268	0.397
40	0.306	0.257
41	0.259	0.363
42	0.226	0.340
43	0.219	0.359

Correlation coefficient^b^	0.534*	

^a^Guyatt’s responsiveness index.

^b^Correlation coefficient between WURSS-K and WURSS.

*P < 0.001.

## DISCUSSION

Reliability refers to the consistency of a result, ie, if the scores from one experiment can be trusted.^25^ Internal reliability defines the consistency of the results delivered in a test, ensuring that the various items measuring different constructs yield consistent scores.^30^ Internal reliability can be measured between similar items in the same domain within the same test. In the internal reliability test, most domains of the WURSS-K had Cronbach’s alphas indicating respectable or very good reliability. The smallest alpha was 0.666, for the “aches” domain. A potential reason for this result is that it includes 2 items, “body aches” and “swollen glands,” which are not closely related. Similarly, the “aches” domain showed low consistency in the WURSS study^21^ (0.624). In addition, the correlation coefficient between the WURSS-K and WURSS was 0.710 (*P* = 0.02),^21^ which means that the WURSS-K and WURSS showed a similar trend in reliability coefficients across domains. In the development of an abbreviated version of the WURSS-K (similar to the WURSS-21, which used only 21 of the 44 items on the WURSS to improve its responsiveness^21^), the “aches” domain and domains with a coefficient alpha greater than 0.90 might be cancelled or adjusted.

The convergent validity did not meet expectations because the Pearson correlation between the 4-week recall of SF-8 and the WURSS-K was −0.267 (*P* = 0.007), indicating a low correlation. This convergent validity was lower than that between the 24-hour recall of SF-8 and the WURSS, which ranged from −0.60 to −0.84 (*P* < 0.001) from day 2 through day 5. This discrepancy is attributable to differences in the recall time of the SF-8, ie, 4-week recall (present study) vs 24-hour recall (WURSS study), the small number of participants, and the narrow age range of the participants enrolled in this research. The evaluation of convergent validity is also limited because it was compared only with the SF-8. Participants from a broader range of age groups and additional instruments such as the Jackson scale will be necessary in future studies.

As for responsiveness, the maximum was 0.46, for the first item, which concerned global condition and was thus expected to display maximum responsiveness. The second maximum was 0.38, for the 32nd item (tiredness), and the minimum value was 0.13, for the 21st item (sinus pressure). The correlation coefficient between responsiveness of the WURSS-K and WURSS was 0.534 (*P* < 0.001), which indirectly proves that the WURSS-K sensitively reflected changes in symptoms.

In summary, the Korean translation of the WURSS was almost equivalent to the WURSS in reliability, validity, and responsiveness and is therefore appropriate for assessing the severity of the common cold in Korean patients.
